# The Mental Health of Older Veterans Ages 58-99 Years: 2016-2017 VE-HEROeS Findings

**DOI:** 10.1093/geroni/igaa057.551

**Published:** 2020-12-16

**Authors:** Yasmin Cypel, Paula Schnurr, Robert Bossarte, William Culpepper, Aaron Schneiderman, Fatema Akhtar, Sybil Morley, Victoria Davey

**Affiliations:** 1 U.S. Department of Veterans Affairs, Washington, District of Columbia, United States; 2 U.S. Department of Veterans Affairs, White River Junction, Vermont, United States; 3 West Virginia University, Morgantown, West Virginia, United States; 4 U.S. Department of Veterans Affairs, Canandaigua, New York, United States

## Abstract

Mental health and its correlates were examined in U.S. Vietnam War veterans approximately fifty years after the War. The 2016-2017 VE-HEROeS (Vietnam Era Health Retrospective Observational Study) was a mail survey of the health of U.S. Vietnam War veterans who served between February 28, 1961 and May 7, 1975 and matched US non-veteran controls. ‘Veteran status’ represented wartime experience for three cohorts: ‘theater’ veterans with service in Vietnam, Cambodia, or Laos, non-theater veterans with service elsewhere, and non-veterans with no military service. Veterans and non-veterans, aged 58-99 years, were randomly selected from a veteran sampling frame (n=9.87 million) derived from the Department of Veterans Affairs’ USVETS dataset and a commercial address database, respectively. Questionnaires were mailed to 42,393 veterans and 6,885 non-veterans; the response rate for veterans was 45% (n=18,866) and 67% (n=4,530) for non-veterans. Weighted bivariate and multivariable analyses were conducted to examine poor overall mental health, via the SF-8TM Mental Health Component Summary score (MCS), and other mental health measures by veteran status and socioeconomic, health, and other military characteristics. Nearly 50% of all theater veterans reported poor overall mental health (MCS<50). Prevalence of mental health measures was greatest for theater veterans and successively decreased for non-theater veterans and non-veterans. Key correlates significantly (P< 0.02) associated with poor MCS included veteran status, race/ethnicity, income, physical health, health perception, trauma, distress, depression, posttraumatic stress disorder (Primary Care DSM-5 PTSD screen), and drug use. Results indicate a high burden of poor mental health among those who served in-theater.

